# Darwinian Selection Discriminates Young Athletes: the Relative Age Effect in Relation to Sporting Performance

**DOI:** 10.1186/s40798-021-00300-2

**Published:** 2021-03-01

**Authors:** Johan Jakobsson, A. Lennart Julin, Glenn Persson, Christer Malm

**Affiliations:** 1 Section for Sports Medicine, Department of Community Medicine and Rehabilitation, Umeå University, Gösta Skoglunds väg 3, SE-901 87 Umeå, Sweden; 2 Swedish Athletic Association, 120 30, Heliosgatan 3, Stockholm, Sweden

**Keywords:** Relative age effect, Sports, Participation, Youth, Athletes, Health

## Abstract

**Background:**

The relative age effect (RAE) is a worldwide phenomenon, allowing sport participation and elite selection to be based on birthdate distribution. Negative consequences include both a narrow, non-optimal elite selection and negative health effects on entire populations. This study investigated the RAE and athletic performance in multiple individual sports in Sweden.

**Methods:**

Birthdates of athletes born between the years 1922 and 2015 were collected across 4-month periods (tertiles: T1, T2, T3) from cross-country skiing (*N* = 136,387), orienteering (*N* = 41,164), athletics (*N* = 14,503), alpine skiing (*N* = 508), E-sports (*N* = 47,030), and chess (*N* = 4889). In total, data from 244,560 athletes (women: *N* = 79,807, men: *N* = 164,753) was compared to the complete parent population of 5,390,954 births in Sweden during the same years. Chi-squared statistics compared parent and cohort distributions stratified by sport, sex, and age.

**Results:**

A significantly skewed distribution of birthdates was present in all sports, both sexes, and most age groups. The largest RAEs are seen in children where T1 often constitutes 40–50% and T3, 20–25% of the population. In E-sports, an inversed RAE was seen in adults. In most investigated sports, birthdate distribution was correlated to performance in children but not in adults.

**Conclusions:**

Skewed birthdate distributions were consistently prevalent in all investigated individual sports in Sweden, both physically demanding and cognitive/skill-based. As sport participation is related to total level of physical activity, both present and future, failing to address the RAE issue at an early age will result not only in a narrow and arbitrary selection for adult elite athletes but also in a negative impact on public health.

**Supplementary Information:**

The online version contains supplementary material available at 10.1186/s40798-021-00300-2.

## Key Points

- The relative age effect is consistently prevalent in most individual sports in Sweden, both physically demanding and cognitive/skill-based.
- In most sports, earlier born children perform better and are higher ranked than later-born peers, a trend not seen in adult athletes where there is no clear correlation between birth date and performance.
- Failing to address the relative age issue at an early age will result not only in a narrow, arbitrary, selection for adult elite athletes, but also in a negative impact on public health.

## Introduction

Selection of future athletes at a young age favors birthdates early in the season, creating a skewed distribution also among adult elite athletes in team sports [1]—the relative age effect (RAE). Human development related to date of birth was described already in the 1920s [2] and further described in the 1930s [3]. Later, RAE was described in the context of academic performance [4] and mental illness [5]. In 1982, “The unknown exclusion in sports” published in “Athletics” (Swedish) first revealed the RAE in sports [6]. Today, RAE is a well-known and worldwide phenomenon, influencing the acquisition of young athletes for future elite performance [7], but children with elite-level ambition and potential, born late in the season, have an unjustified lower chance of sport participation past puberty [8].

Relative age effects are apparent across multiple sports, including ice hockey [9–11], soccer [12–14], baseball [15], basketball [13, 16], American football [17], rugby [18, 19], handball [20], swimming [21], and tennis [22]. In gymnastics [23] and athletics [24], reversed RAEs have been described. Further, RAEs are prevalent across all age groups from children to adults, and all skill levels [19]. In team sports, RAEs are unanimous across countries and persists into adult elite squads [14, 25], such as in males drafted for the NHL 2000–2005 [26] and in 1997–2007 FIFA U17 World Cup players, where 40% were born in the first, and only 16% in the last, quarter of the year [27]. Significant RAEs in male professional soccer players were also found in Italy [28], Germany, Japan, Brazil, and Australia [29]. While common, some argue that RAEs are not always present, especially not in women’s sports [30].

When present on a larger scale, negative health effects of early selection in sports are inevitable, as an unhealthy chain of events is initiated: As an increasing proportion of physical activity takes place in organized forms [31], lack of sport participation, as a result of RAE selection when young, will thus reduce the lifetime levels of physical activity [32] and subsequently increase the risk of inactivity-related diseases.

Another problematic issue of biased selection in sports is the violation of the guiding principles by the United Nations Convention on the Rights of the Child (CRC) [33]. Here, Article 2 states the equal value of all children and that every child has the right to participate. Further, Article 3 states that all decisions concerning a child should be made in the best interest of the child. As of January 1, 2020, CRC is legally binding in Sweden [34].

To ensure fair and equal competition, youth sports are generally organized into age groups by date of birth [14, 35], ranging over a 1- to 2-year period with cut-off times differing between sports and countries [14, 36]. This organization, with a full 2-year age difference within the same age group, and even more in biological maturation, is one aspect of the RAE [7, 14]. Other social agents, further described by Hancock, Adler [37], have also been proposed to have large influences on RAEs. Age-group division benefits relatively older athletes, while increasing the risk of injury and early dropout from sports among children born later in the season [7, 18, 38]. The highest RAE prevalence is found in team sports [35], but exists also in individual sports such as alpine skiing and Nordic combined [39], swimming [40], master swimming and athletics [41], shooting [42], ski jumping, and snowboarding [39]. Sports with high physical, rather than technical demands are more often affected [25, 43].

Our understanding of RAEs in individual sports is limited, and previous findings need validation by studies with adequate sample size [39]. Published studies have rarely considered the parental population birthdate distribution [13, 35, 39, 40] but assumed equal distribution (25% per quarter and 0.083% per month) [39, 44], sometimes including the day-corrected statistic [45]. Using the parental population as comparison is key in chi-squared tests [46], else the Type 1 error is not controlled for and the goodness-of-fit test biased.

The purpose of the present study was twofold: (1) to examine the RAE in individual sports, including both physically demanding (cross-country skiing, alpine skiing, athletics, orienteering) and cognitive/skill-based (E-sports and chess) sports and (2) to investigate the relationship between RAE and performance.

## Method

### Procedure

Birthdate data was collected from official websites or provided by the corresponding sport’s federation. Cross-country skiing data was retrieved from three different datasets: From the page of FIS (http://fisski.com) and the Swedish Ski Federation (http://skidor.com) and kindly provided by Johan Nyman at http://skidresultat.se. The three datasets contain both overlapping and unique data and were therefore not merged but analyzed separately (Table 1). Alpine skiing data was retrieved from FIS (http://fisski.com), and athletics data was provided by Bo Nordin and A. Lennart Julin, retrieved from an internal database of Swedish athletics results. These results include the annual best performance (top 20 to 30, depending on year and event) for all age groups. Orienteering data was retrieved from the Swedish Orienteering federation (http://www.svenskorientering.se/), Chess data from the Swedish Chess Federation (https://schack.se/), and E-sports data from Swedish Gaming Association (https://sverok.se/). Athletes whose birthdates were not declared by the dataset were excluded from the study. Duplicates were removed (participation in more than one age group or event) leaving us with a total of 244,558 individuals.

**Table 1:** Data overview

Sport	Data retrieved	Age	Age range	Total ***N***	Females	Males
Cross-country skiing^a^	2014	33.6 ± 19.8	1–90	124,139	50,684	73,381
Cross-country skiing^b^	2015	34.9 ± 17.2	4–90	11,377	2596	8781
Cross-country skiing^c^	2015	21.6 ± 4.5	17–39	943	361	582
Alpine Skiers	2015	18.9 ± 3.2	17–39	502	244	258
Athletics	2019	21.8 ± 9.8	10–68	14,503	7511	6992
Orienteering	2015	34.7 ± 21.2	1–95	41,164	16,925	24,239
Chess	2016	41.4 ± 22.3	5–95	4900	327	4562
E-Sport	2016	21.7 ± 5.2	10–45	47,030	1159	45,871
All athletes	–	31.0 ± 18.5	1–95	244,558	79,807	164,666
Reference population	2016	–	–	5,390,954	2,618,818	2,772,136

^a^Dataset retrieved from the official timing system for Swedish Skiing “SSF-Timing” and includes recreational, competitive, and elite skiers entered in one or more competitions sanctioned by the Swedish Ski Association

^b^Dataset retrieved from www.skidresultat.se, and includes recreational, competitive, and elite skiers finishing one or more races

^c^Dataset of FIS-registered (elite) cross-country skiers. In total, the sex of 74 subjects was unspecified. Reference population refers to all births in Sweden between the years 1961 and 2011

### Data collection

Birthdates were summarized across 4-month periods (hereafter called tertiles, T) as described in Table 1. Subsequently, tertile one (T1) includes athletes with birthdates from January 1st to April 28th (or 29th), tertile two (T2) from May 1st to August 31st, and tertile three (T3) from September 1st to December 31st. Age distribution summarized in Table 2. We chose to divide the year into tertiles instead of the more common quarters, as both divisions are arbitrarily time-periods, with tertiles giving higher statistical power.

**Table 2:** Age distribution of whole athlete population

Age	***N***
≤ 6	3528
7–8	7336
9–10	11,125
11–15	33,267
16–20	41,316
21–39	66,230
≥ 40	81,756

As a reference parent population, the complete record of all Swedish births between the years 1961 and 2011 was retrieved from Statistics Sweden (https://www.scb.se/) and used for all chi-squared analyses, with exact adjustment for each single analysis. In the reference parent population, birth distributions were T1 = 35.1%, T2 = 34.5%, and T3 = 30.4% (Supplementary file 1). For analyses including individuals mainly or only born earlier than 1961, the birth distribution of 1961-1970 was assumed (T1 = 35.4%, T2 = 33.6%, T3 = 31%).  

### Stratified Analyses

Analyses were executed for the whole sample and divided into age groups’ sport type (physical and skill-based sports). To determine the effect of age on RAEs, samples were categorized into children (≤ 10 years), young adolescent (11–15 years), adolescents (16–20 years), adults (21–39 years), and masters (40 years and older). Where applicable (*N* ≥ 70), further sub-division were made including ≤ 6 years, 7–8 years, 9–10 years, 40–59 years, and ≥ 60 years. Data was sub-analyzed by sport (three different datasets covering cross-country skiing) and sex. For athletics, data includes athletes competing in the age groups from 14 years and older and seen as juniors until 23 years of age. Thus, for athletics, age groups were 14–17 years, 18–22 years, and 23 years and older. For all sports, except athletics, sub-disciplines (i.e., skiing, or orienteering distance, alpine discipline) are not specified. For athletics, sub-analyses are executed for each event, and by sex and age when adequate. When not specified, all events are included in the “athletics” analysis.

When available, ranking (orienteering), rating, (cross-country skiing dataset 2 and chess), and performance (athletics) data were analyzed as well.

### Statistical Analysis

A cut-off date on Jan 1st was used, which is the standard procedure in all Swedish sports. Chi-squared tests assessed differences in frequency counts between tertiles. Estimated distribution with 95% confidence interval (CI) is reported. The parent population distribution was set as hypothesized probability, adjusted for each chi-squared analysis. Cramer’s *V* (*V*) is calculated as the effect size (ES) and interpreted as a small (0.07), medium (0.21), or large effect (0.35) [47]. Distribution analyses were not executed on sub-divided samples (i.e., a certain age group and/or sex in a certain sport/event) with less than 70 individuals. Because the parental population (expected) distribution is known, the ratio between expected and observed distributions (hereinafter called ratio) is expressed in all figures for visualization purposes. Because the parent distribution (full population) is rarely known, it is common to use odds ratio (OR). We chose to use the expected (true) versus observed distribution ratio, as we indeed had the full, parental dataset of the population. Also, compared to OR this ratio is easier to understand by the non-statistician.

Rating data (i.e., higher is better in cross-country skiing and chess) and ranking data (i.e., lower is better in orienteering) were analyzed using the non-parametric Kruskal-Wallis one-way analysis of variance test with the Steel-Dwass method for post-hoc comparisons. Performance data (athletics) was analyzed using one-way analysis of variance (ANOVA), with Tukey’s honest significant difference test for post-hoc comparisons. Mean and standard deviation were used for parametric descriptive statistics, and median with interquartile range (IQR) for non-parametric data. All calculations were made in JMP 15.1 (SAS Institute, USA) and statistical significance set at *P* ≤ 0.05.

## Results

In all investigated sports both sexes show significantly skewed birthdate distribution, starting at age groups 6 years and younger.

Due to a large amount of data, results are herein summarized. Complete statistical data can be found in supplementary materials: reference population raw data (Supplementary file 1), distribution analyses with chi-squared statistics (2), expected to observed distribution ratios (3), performance and rating/ranking (4), and raw data for all athlete datasets (5).

### Distribution of Birthdates Among Athletes: Summarized

**Fig. 1:**
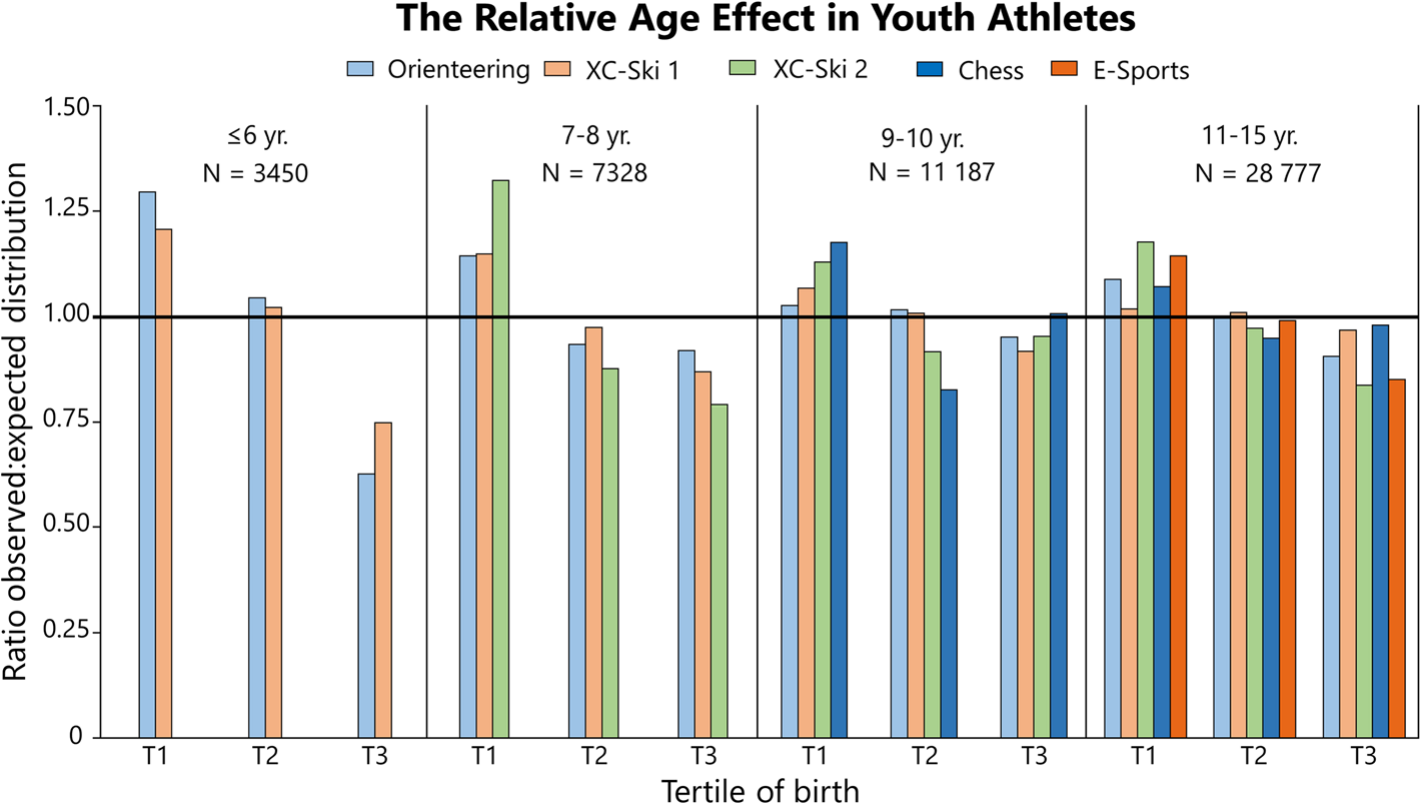
The relative age effect expressed as the ratio between observed and expected distributions (national birth statistics) of athletes per tertile (T). XC-Ski 1 refers to dataset cross-country skiing^1^ and XC-Ski 2 refers to dataset cross-country skiing^2^. For additional data, see Supplementary file 2

**Fig. 2:**
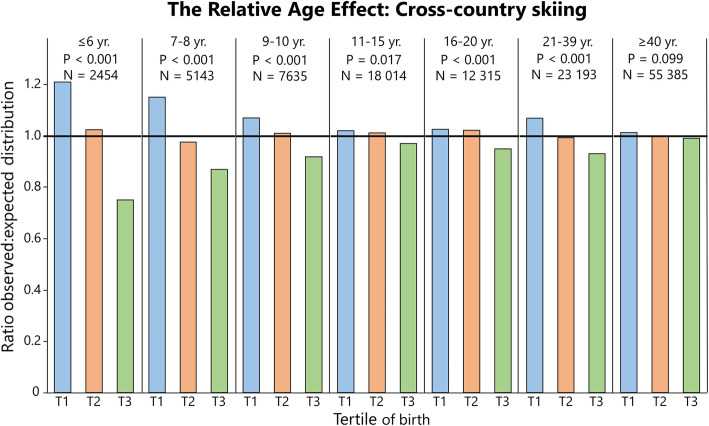
The relative age effect in Swedish cross-country skiers expressed as the ratio between observed and expected distributions (national birth statistics) of athletes per tertile (T). Data refers to cross-country skiing^1^*.* For additional data, see Supplementary file 2

**Fig. 3.**
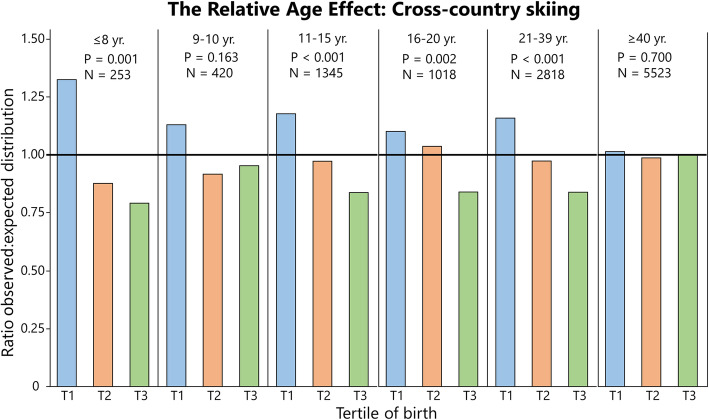
The relative age effect in Swedish cross-country skiers expressed as the ratio between observed and expected distributions (national birth statistics) of athletes per tertile (T). Data refers to cross-country skiing^2^. For additional data, see Supplementary file 2

**Fig. 4:**
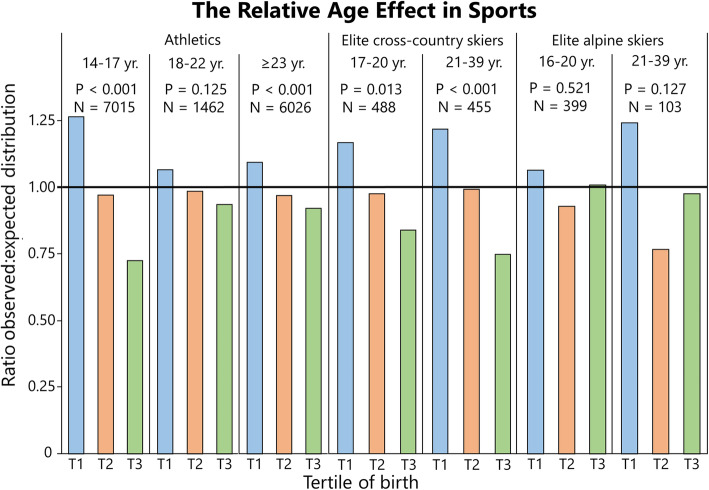
The relative age effect in Swedish athletic athletes, elite cross-country skiers (dataset cross-country skiing^3^ and elite alpine skiers) expressed as the ratio between observed and expected distributions (national birth statistics) of athletes per tertile (T). For additional data, see Supplementary file 2

**Fig. 5:**
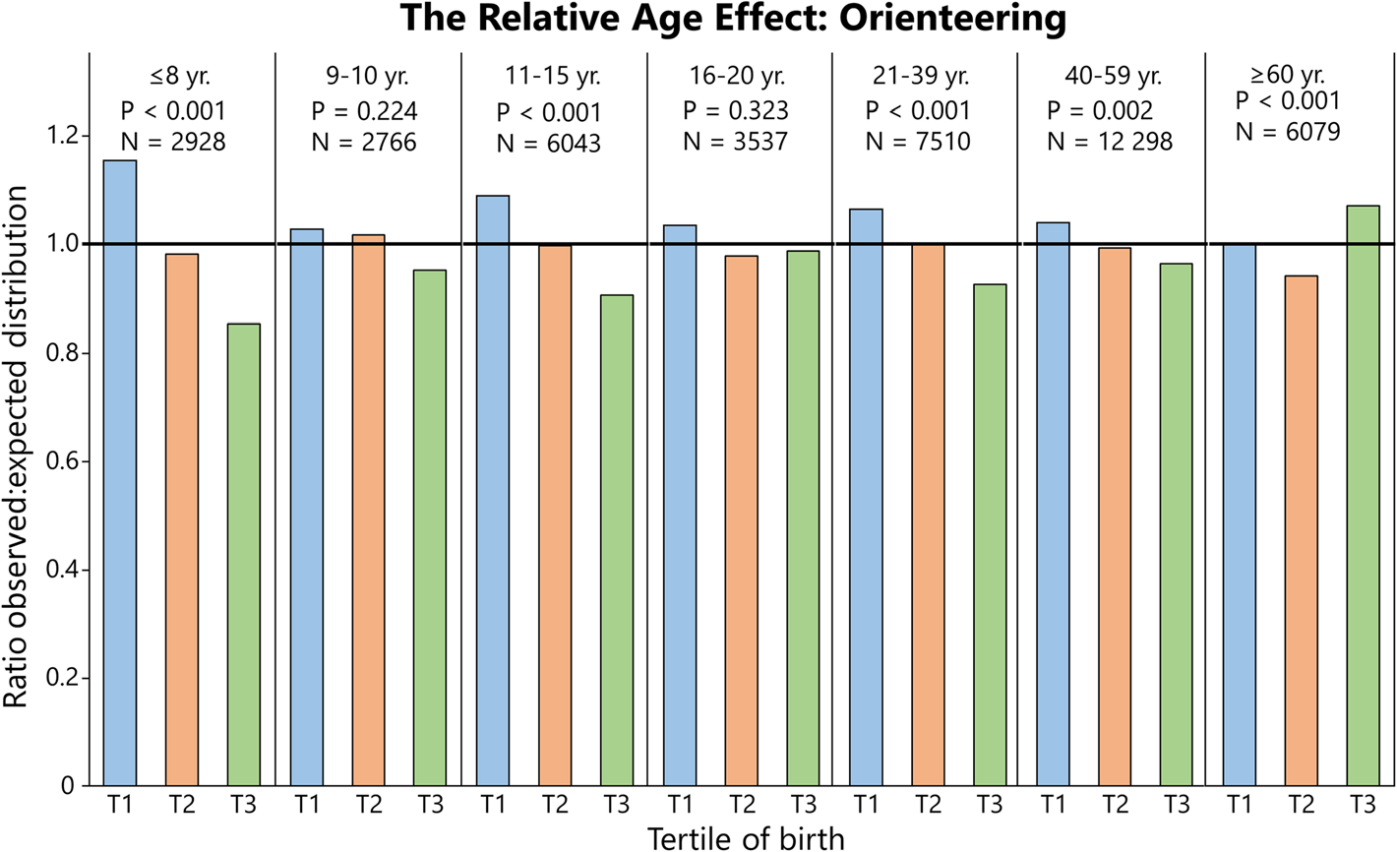
The relative age effect in Swedish orienteers expressed as the ratio between observed and expected distributions (national birth statistics) of athletes per tertile (T). For additional data, see Supplementary file 2

**Fig. 6:**
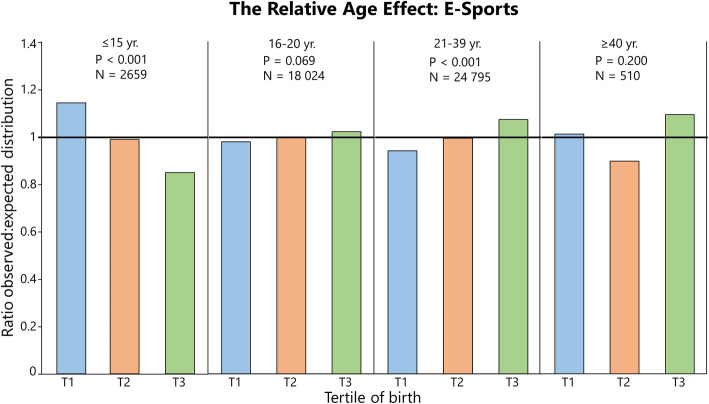
The relative age effect in Swedish E-sports players expressed as the ratio between observed and expected distributions (national birth statistics) of athletes per tertile (T). Age groups ≤ 15, 16–20, and 21–39 years include males only. For additional data, see Supplementary file 2

**Fig. 7:**
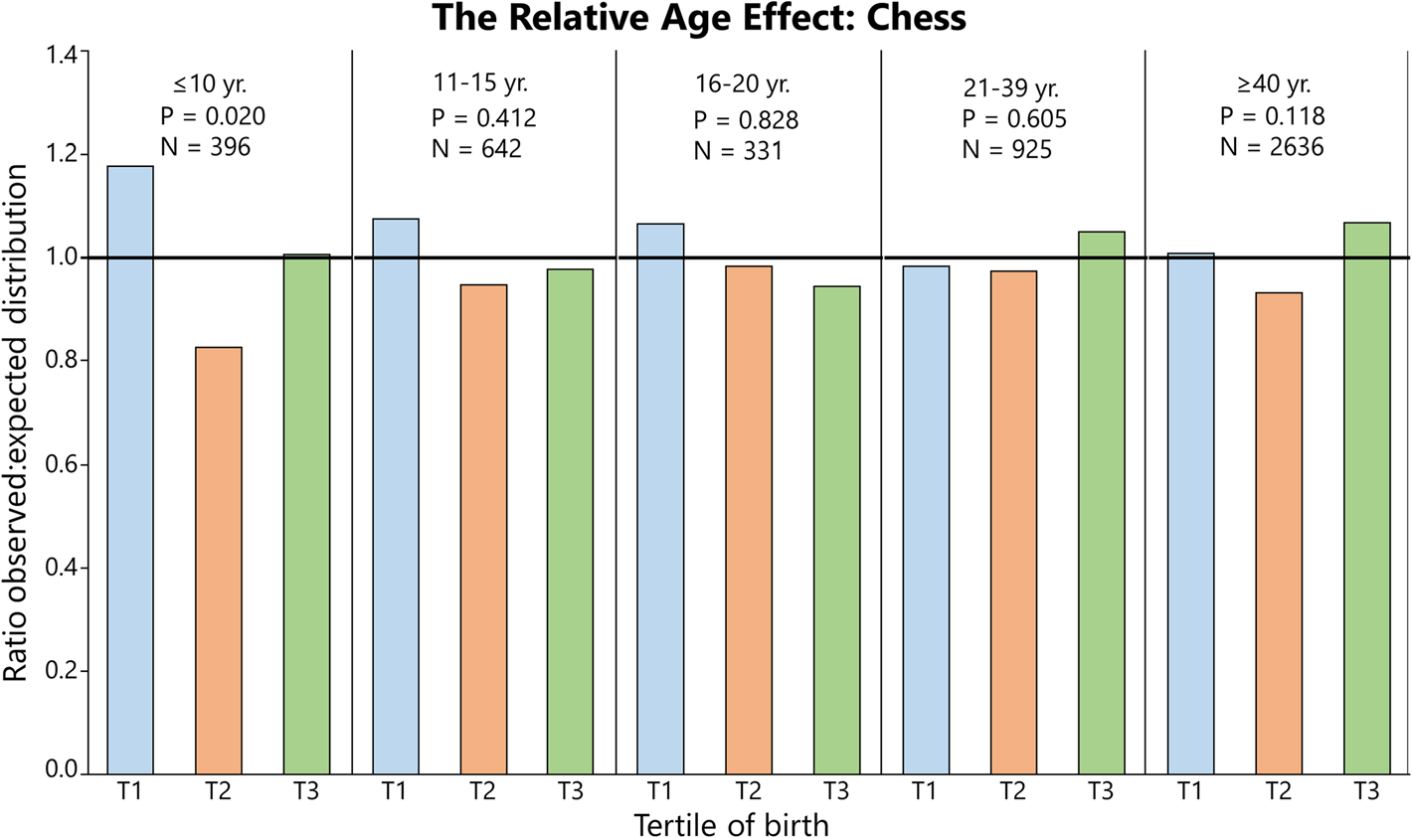
The relative age effect in Swedish chess players expressed as the ratio between observed and expected distributions (national birth statistics) of athletes per tertile. For additional data, see Supplementary file 2

Table 3 shows the distribution statistics for all sports and ages pooled, analyzed by sport. Here, RAEs are evident in the whole population of athletes and in physical sports alone, while in skill-based sports, this is true for males but not females. Table 4 shows the distribution of athletes sub-analyzed by sport, and for two specific events in athletics. Table 5 shows the distribution analyzed by age group and sex of the whole sample. Relative age effects are present in all age groups except for males in the age of 21–39 years, and adults over the age of 40 years. Figures 1, 2, 3, 4, 5, 6 and 7 show the tertile distribution, expressed as the ratio between true and observed frequencies, in analyzed sports and age groups. Details for sub-analyses by sex, age group, sport, and event are found in supplementary file 2.

**Table 3:** Distribution and summary statistics for the whole athlete population and by sport type

Sport	Sex	Chi-squared statistics		Absolute distribution (***N***)				Relative distribution (%) (95% CI)			
		***Χ***^**2**^	***P*** value	Total	T1	T2	T3	T1	T2	T3	***V***
All	Both	205.0	< 0.001	244 558	89 036	83 781	71 741	36.4 (36.2–36.6)	34.3 (34.1–34.4)	29.3 (29.2–29.5)	0.12
	Male	61.1	< 0.001	164 666	59 269	56 492	48 905	36.0 (35.8–36.2)	34.3 (34.1–34.4)	29.7 (29.5–29.9)	0.10
	Female	192.9	< 0.001	80 077	29 752	27 521	22 804	37.3 (36.9–37.6)	34.1 (33.8–34.5)	28.6 (28.3–28.9)	0.16
Physical	Both	2127.0	< 0.001	192 628	71 052	65 965	55 611	36.9 (36.7–37.1)	34.3 (34.0–34.5)	28.9 (28.7–29.1)	0.23
	Male	135.0	< 0.001	114 233	41 827	39 173	33 233	36.6 (36.3–36.9)	34.3 (34.0–34.6)	29.1 (28.9–29.4)	0.13
	Female	194.4	< 0.001	78 321	29 212	26 760	22 349	37.3 (37.0–37.6)	34.2 (33.8–34.5)	28.5 (28.2–28.9)	0.16
Skill	Both	12.7	0.002	51 930	17 984	17 816	16 130	34.6 (34.2–35.0)	34.3 (33.9–34.7)	31.1 (30.7–31.5)	0.09
	Male	13.2	0.001	50 433	17 442	17 319	15 672	34.6 (34.2–35.0)	34.3 (33.9–34.8)	31.1 (30.7–31.5)	0.09
	Female	1.6	0.442	1 486	540	491	455	36.3 (34.0–38.8)	33.0 (30.7–35.5)	30.6 (28.3–33.0)	0.13

*Χ*^2^ chi-squared statistic, *T* tertile, *V* effect size, Cramer’s *V*

Parent population (national birth statistics) was set at expected distribution in chi-squared test

“Physical” includes Cross-country skiing, athletics, orienteering and alpine skiing. “Skill” includes E-sports and chess

**Table 4:** Distribution and statistics summary by sport and selected athletic events

Sport	Sex	Chi-squared statistics		Absolute distribution (***N***)				Relative distribution (%) (95% CI)			
		***Χ***^**2**^	***P*** value	Total	T1	T2	T3	T1	T2	T3	***V***
Cross-country skiing^a^	Male	47.0	< 0.001	73 381	26 521	25 330	21 530	36.1 (35.8–36.5)	34.5 (34.2–34.9)	29.3 (29.0–29.7)	0.11
	Female	61.2	< 0.001	50 684	18 571	17 369	14 744	36.6 (36.2–37.1)	34.3 (33.9–34.7)	29.1 (28.7–29.5)	0.13
Cross-country skiing^b^	Male	23.2	< 0.001	8781	3297	2948	2 536	37.5 (36.5–38.6)	33.6 (32.6–34.6)	28.9 (27.9–29.8	0.16
	Female	41.9	< 0.001	2596	1055	875	666	40.6 (38.8–42.5)	33.7 (31.9–35.5)	25.7 (24.0–27.4)	0.25
Cross-country skiing^c^	Male	14.4	0.007	582	243	202	137	41.7 (37.7–45.7)	34.8 (31.1–38.8)	23.5 (20.2–27.1)	0.28
	Female	8.9	0.012	361	153	120	88	42.5 (37.6–47.7)	33.1 (28.5–38.2)	24.3 (20.2–29.0)	0.28
All athletics events	Male	136.3	< 0.001	6992	2861	2387	1744	40.9 (39.8–42.1)	34.1 (33.0–35.3)	24.9 (23.9–26.0)	0.26
	Female	145.1	< 0.001	7511	3108	2494	909	41.4 (40.3–42.5)	33.2 (32.1–34.3)	25.4 (24.4–26.4)	0.26
Athletics 100 m	Male	81.8	< 0.001	1266	584	416	266	46.1 (43.4–48.9)	32.9 (30.3–35.5)	21.0 (18.9–23.3)	0.36
	Female	55.1	< 0.001	976	444	320	212	45.5 (42.4–48.6)	32.8 (29.9–35.8)	21.7 (19.2–24.4)	0.34
Athletics discus	Male	28.5	< 0.001	664	271	252	141	40.8 (37.1–44.6)	38.0 (34.3–41.7)	21.2 (18.3–24.5)	0.32
	Female	19.7	< 0.001	714	303	237	174	42.4 (38.9–46.1)	33.2 (29.8–36.7)	24.4 (21.4–27.7)	0.29
Orienteering	Male	15.8	< 0.001	24 239	8806	8226	7207	36.3 (35.7–36.9)	33.9 (33.3–34.5)	29.7 (29.2–30.3)	0.11
	Female	28.0	< 0.001	16 925	6231	5824	4870	36.8 (36.1–37.6)	34.4 (33.7–35.1)	28.8 (28.1–29.5)	0.14
Alpine skiing	Male	2.0	0.367	258	99	80	79	38.1 (32.7–44.4)	31.0 (25.7–36.9)	30.6 (25.3–36.5)	0.21
	Female	1.4	0.491	244	94	78	72	38.5 (32.6–44.8)	32.0 (26.4–38.1)	29.5 (24.2–35.5)	0.20
E-Sport	Male	11.4	0.003^d^	45 871	15 793	15 848	14 230	34.4 (34.0–34.9)	34.5 (34.1–34.9)	31.0 (30.6–34.0)	0.09
	Female	2.0	0.373	1159	421	382	356	36.3 (33.6–39.1)	33.0 (30.3–35.7)	30.7 (28.1–33.4)	0.14
Chess	Male	10.8	0.005	4562	1649	1471	1442	36.1 (34.8–37.6)	32.2 (30.9–33.6)	31.6 (30.3–33.0)	0.16
	Female	0.3	0.86	327	119	109	99	36.4 (31.4–41.8)	33.3 (28.4–38.6)	30.3 (25.6–35.5)	0.12

***X***^2^ chi-squared statistic, *V* effect size, Cramer’s *V*

Parent population (national birth statistics) was set at expected distribution in chi-squared test

^a^Database retrieved from “SSF-Timing” and includes recreational, competitive and elite skiers

^b^Data retrieved from www.skidresultat.se, and includes recreational, competitive and elite skiers

^c^Dataset of FIS-registered (elite) cross-country skiers

^d^Inversed RAE

**Table 5:** Distribution and statistics summary by age group, sex, and birth tertile

Age	Sex	Chi-squared statistics		Absolute distribution (***N***)				Relative distribution (%) (95% CI)			
		***Χ***^**2**^	***P*** value	Total	T1	T2	T3	T1	T2	T3	***V***
≤ 6	Both	129.8	< 0.001	3528	1435	1281	812	40.7 (39.1–42.3)	36.3 (34.7–37.9)	23.0 (21.7–24.4)	0.31
	Male	91.3	< 0.001	1859	757	704	398	40.7 (38.5–43.0)	37.9 (35.7–40.1)	21.4 (19.6–23.3)	0.33
	Female	45.9	< 0.001	1668	678	577	413	40.6 (38.3–43.0)	34.6 (32.3–36.9)	24.8 (22.7–26.9)	0.29
7–8	Both	90.2	< 0.001	7336	2822	2509	2005	38.5 (37.4–39.6)	34.2 (33.1–35.3)	27.3 (26.3–28.4)	0.24
	Male	46.8	< 0.001	3788	1464	1270	1054	38.6 (37.1–40.2)	33.5 (32.0–35.0)	27.8 (26.4–29.3)	0.24
	Female	44.4	< 0.001	3544	1355	1238	951	38.2 (36.6–39.8)	34.9 (33.4–36.5)	26.8 (25.4–28.3)	0.24
9–10	Both	29.8	< 0.001	11 125	3967	3955	3203	35.7 (34.8–36.6)	35.6 (34.7–36.4)	28.9 (28.0–29.6)	0.16
	Male	14.5	< 0.001	5695	2036	2010	1649	35.8 (34.5–37.0)	35.3 (34.1–36.5)	29.0 (27.8–30.1)	0.16
	Female	15.9	< 0.001	5425	1931	1942	1552	35.6 (34.3–36.8)	35.8 (34.5–37.1)	28.6 (27.4–29.8)	0.17
11–15	Both	232.1	< 0.001	39 068	14 447	13 755	10 866	37.0 (36.5–37.5)	35.2 (34.7–35.7)	27.8 (27.4–28.3)	0.20
	Male	119.9	< 0.001	20 998	7739	7416	5843	36.9 (36.2–37.5)	35.3 (34.7–36.0)	27.8 (27.2–28.5)	0.19
	Female	113.3	< 0.001	18 064	6708	6336	5020	37.1 (36.4–37.8)	35.1 (34.4–35.8)	27.8 (27.1–28.5)	0.20
16–20	Both	18.2	< 0.001	41 316	14 873	14 574	11 869	36.0 (35.5–36.5)	35.3 (34.8–35.7)	28.7 (28.3–29.2)	0.10
	Male	40.6	< 0.001	30 363	10 793	10 749	8821	35.5 (35.0–36.1)	35.4 (34.9–35.9)	29.1 (28.5–29.6)	0.14
	Female	49.9	< 0.001	10 944	4077	3819	3048	37.3 (36.5–38.2)	34.9 (34.0–35.8)	27.8 (27.0–28.7)	0.18
21–39	Both	19.0	< 0.001	66 230	24 345	22 441	19 444	36.8 (36.4–37.1)	33.9 (33.5–34.2)	29.3 (29.0–29.7)	0.09
	Male	0.8	0.686	49 220	17 715	16 746	14 759	36.0 (35.6–36.4)	34.0 (33.6–34.4)	30.0 (29.6–30.4)	0.04
	Female	76.7	< 0.001	16 995	6627	5691	4677	39.0 (38.3–39.7)	33.5 (32.8–34.2)	27.5 (26.9–28.2)	0.18
≥ 40	Both	3.5	0.174	81 756	29 173	27 383	25 200	35.7 (35.4–36.0)	33.5 (33.2–33.8)	30.8 (30.5–31.1)	0.06
	Male	1.3	0.536	55 721	19 813	18 669	17 239	35.6 (35.2–36.0)	33.5 (33.1–33.9)	30.9 (30.6–31.3)	0.05
	Female	4.3	0.114	25 987	9354	8691	7942	36.0 (35.4–36.6)	33.4 (32.8–34.0)	30.6 (30.0–31.1)	0.08
All	Both	205.0	< 0.001	244 558	89 036	83 781	71 741	36.4 (36.2–36.6)	34.3 (34.1–34.4)	29.3 (29.2–29.5)	0.10
	Male	61.1	< 0.001	164 666	59 269	56 492	48 905	36.0 (35.8–36.2)	34.3 (34.1–34.4)	29.7 (29.5–29.9)	0.16
	Female	192.9	< 0.001	80 077	29 752	27 521	22 804	37.3 (36.9–37.6)	34.1 (33.8–34.5)	28.6 (28.3–28.9)	0.12

*Χ*^2^ chi-squared statistic, *V* effect size, Cramer’s *V*

Parent population (national birth statistics) was set at expected distribution in chi-squared test

Stratified by sport (Table 4), RAEs are present in all sports except elite alpine skiing, female E-sports, and chess. However, in E-sporting males, the RAE is inversed in some age groups (21–39) while an ordinary RAE is seen in some (Fig. 6). Regarding age, the magnitude of skewness decreases with higher age, seen in Table 5 and Figs. 1, 2, 3, 4, 5, 6 and 7. Summarized in Fig. 1, RAEs are highly prevalent in young athletes in orienteering, cross-country skiing, chess, and E-sports.

### Distribution of Athletes: By Dataset

As seen in Fig. 2, cross-country skiing^1^ (*N* = 124 139) shows significant RAEs in all age groups, ranging from ≤ 6 to 21–39 years. Effect sizes are largest in the youngest athletes (≤ 6 years, *V* = 0.31) and decrease with higher age (≥ 40 years, *V* = 0.07). Stratified by sex, RAEs exist in all age groups ranging from ≤ 6 to 21–39 (*P* < 0.05) except for 11–15-year-old girls (*P* = 0.200, *V* = 0.1, ratio_T1_ = 1.03).

In Fig. 3, data on cross-country skiing^2^ (*N* = 11 377) is shown, where RAEs are present in both sexes in athletes up to 40 years of age. The most skewed distribution in the whole population was found in boys up to 8 years with 51% (ratio = 1.53), 29% (0.82) and 20% (0.64) of the athletes born in T1, T2, and T3, respectively (Supplements 2 and 3). As seen in Fig. 3, the skewed distribution becomes less evident in older age groups. The effect size was medium (≤ 0.21 V < 0.35) for all age groups up to 21–39 years and small (*V* = 0.07) for ≤ 40 years. Here, a skewed distribution was evident in 11–15-year-old girls (*P* = <0.001, *V* = 0.29, ratio_T1_ = 1.18), which differs from cross-country skiing^1^.

In elite cross-country skiers (Fig. 4), a skewed distribution was found in both junior and senior athletes (Fig. 4) with medium effect sizes (*V* = 0.26 and 0.31, respectively). When sub-analyzed by sex, significance remained only for male seniors (Supplement 2). In elite alpine skiers, no significant RAE was found, neither in junior athletes nor in senior athletes. A sub-analysis by sex shows a significant RAE in adult males, but with limited sample size (*N* = 70).

In athletics (Fig. 4), RAEs were seen in young adolescents (14–17 years, *V* = 0.33) and adults (23 years and older, *V* = 0.18) but not older adolescents (18–22 years, *V* = 0.16) when analyzed by age groups (Fig. 4). For athletics, RAEs were consistently evident in both sexes. The most skewed distribution when analyzed by age group was found in 14-year-old boys with 51% (1.3), 33% (0.98), and 16% (0.68) of the population in T1, T2, and T3, respectively (*V* = 0.45), with similar distribution for girls, 48% (1.24), 33% (0.97), and 19% (0.76), respectively (*V* = 0.41).

Sub-stratified by specific events, the most skewed distributions and largest effect sizes were found in 60 m (children/young adolescents), 100 m, 400 m, high jump, and pole vault while the distribution is not skewed in long-distance running with mainly adult competitors (3000 m–42.2 km). Further, no significant RAE was found in shot put (*N* = 216) or 60-m hurdles (*N* = 156). For additional details on athletic events, see Supplement 2.

In orienteering (Fig. 5), RAEs were found in age groups ≤ 8, 11–15, 16–20, 21–39, 40–59, and ≤ 60 years, with *V* = 0.25, 0.19, 0.11, 0.17, 0.06, 0.13, and 0.16, respectively. Significant RAEs were evident in all age groups including boys and girls up to 5 years of age (*P* < 0.001, *V* = 0.37). In adults over 60 years, while the distribution is near equal (≈ 33% per tertile), an inversed RAE is evident in both sexes (*P* < 0.05, *V* = 0.14, ratio T3 = 1.06, for males; *P* < 0.05, *V* = 0.19, ratio T3 = 1.09 for females) because the parental distribution for corresponding years is not evenly distributed (Supplements 1, 2, and 3).

In E-sports (Fig. 6), RAEs were seen in boys up to 15 years of age (*V* = 0.25). In 21–39-year-old males, an inversed RAE was evident (*V* = 0.17), where age groups ≤ 15, 16–20, and 21–39 years include males only because females constitute of ~ 3% of the total population in those age groups. When the whole E-sports sample is studied, RAEs are evident for males (*P* = 0.034, *V* = 0.09) but not for females (*P* = 0.373, *V* = 0.14) (Table 5). The RAE in male E-sports players was inversed, while this is only significant for 21–39-year-olds (ratio T3 = 1.08) when analyzed by age group.

In chess (Fig. 7), RAEs were significant in children up to 10 years of age (*V* = 0.26) but no other sub-sample when analyzed by age group. As seen in Table 5, a pooled analysis on all chess players shows significant RAEs, when analyzed by sex only in males. The significant RAE is atypical, with T2 consistently being underrepresented, while T1 and T3 being overrepresented (Supplements 2 and 3).

### Rating and Performance by Birth Tertile

**Fig. 8:**
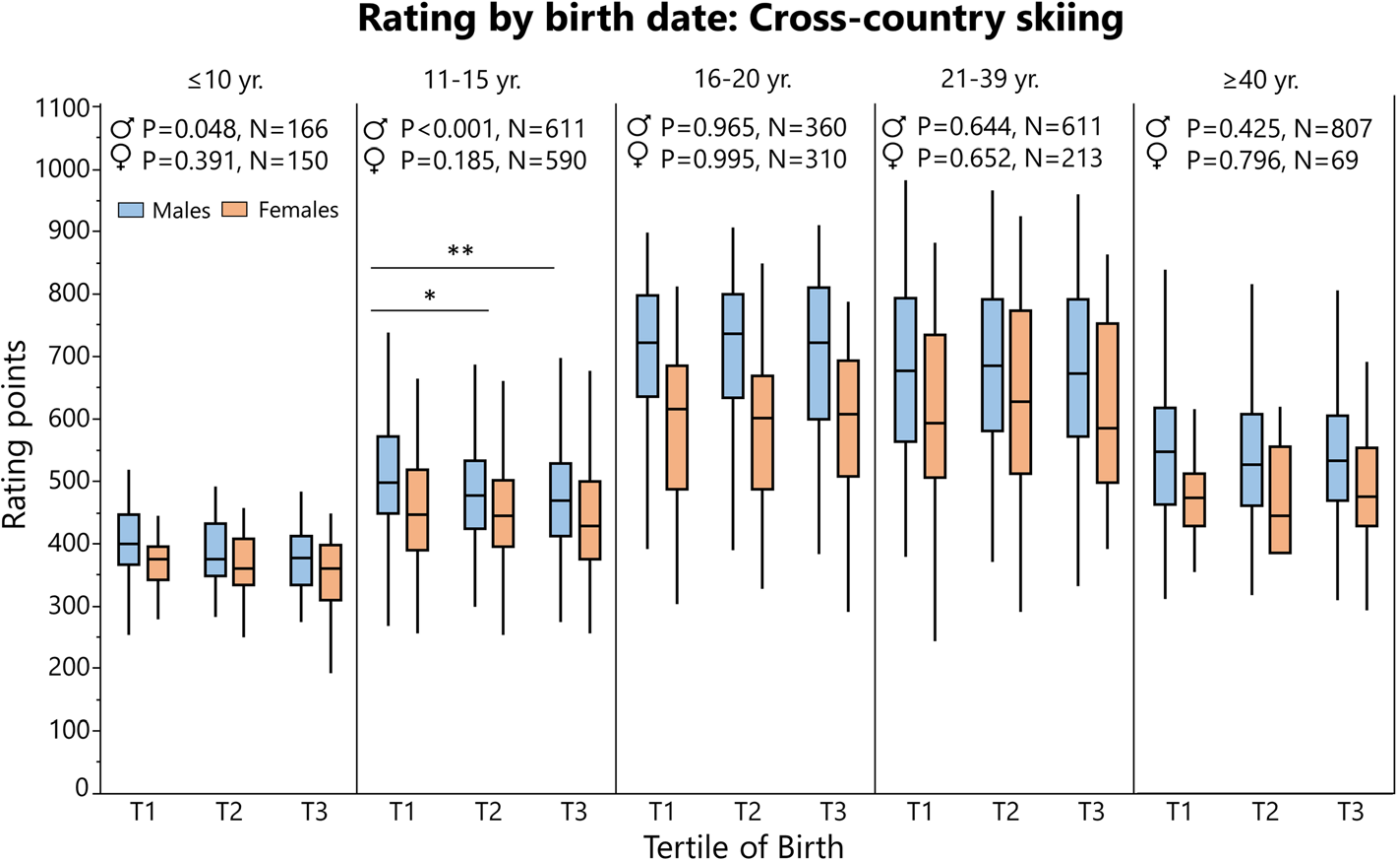
Rating points (i.e., higher is better) for Swedish cross-country skiers analyzed by birth tertile (T) and sex in different age groups with Kruskal-Wallis one-way analysis of variance (statistics at the top of the figure), when significant followed by Steel-Dwass post-hoc test for multiple comparison between groups. The boundary of the box closest to zero indicates the 25th percentile, the line within the box marks the median, and the boundary of the box farthest from zero indicates the 75th percentile. End of whiskers represents the lowest and highest data point within 1.5× interquartile range of the first and third quartile. *P ≤ 0.05, **P ≤ 0.01

**Fig. 9:**
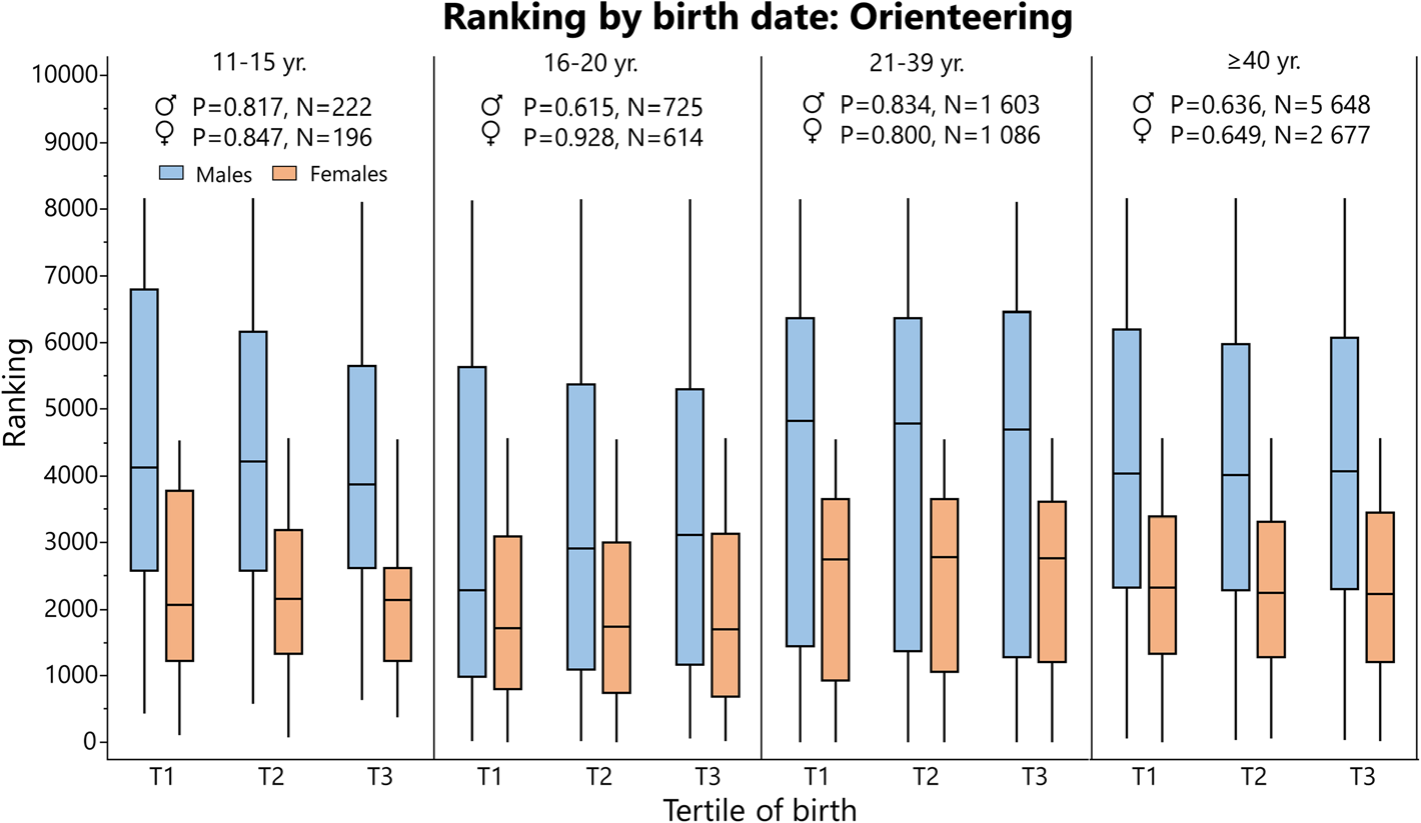
Ranking (i.e., lower is better) for Swedish orienteers analyzed by birth tertile (T) and sex in different age groups with Kruskal-Wallis one-way analysis of variance (statistics at the top of the figure). The boundary of the box closest to zero indicates the 25th percentile, the line within the box marks the median, and the boundary of the box farthest from zero indicates the 75th percentile. End of whiskers represents the lowest and highest data point within 1.5× interquartile range of the first and third quartile

**Fig. 10:**
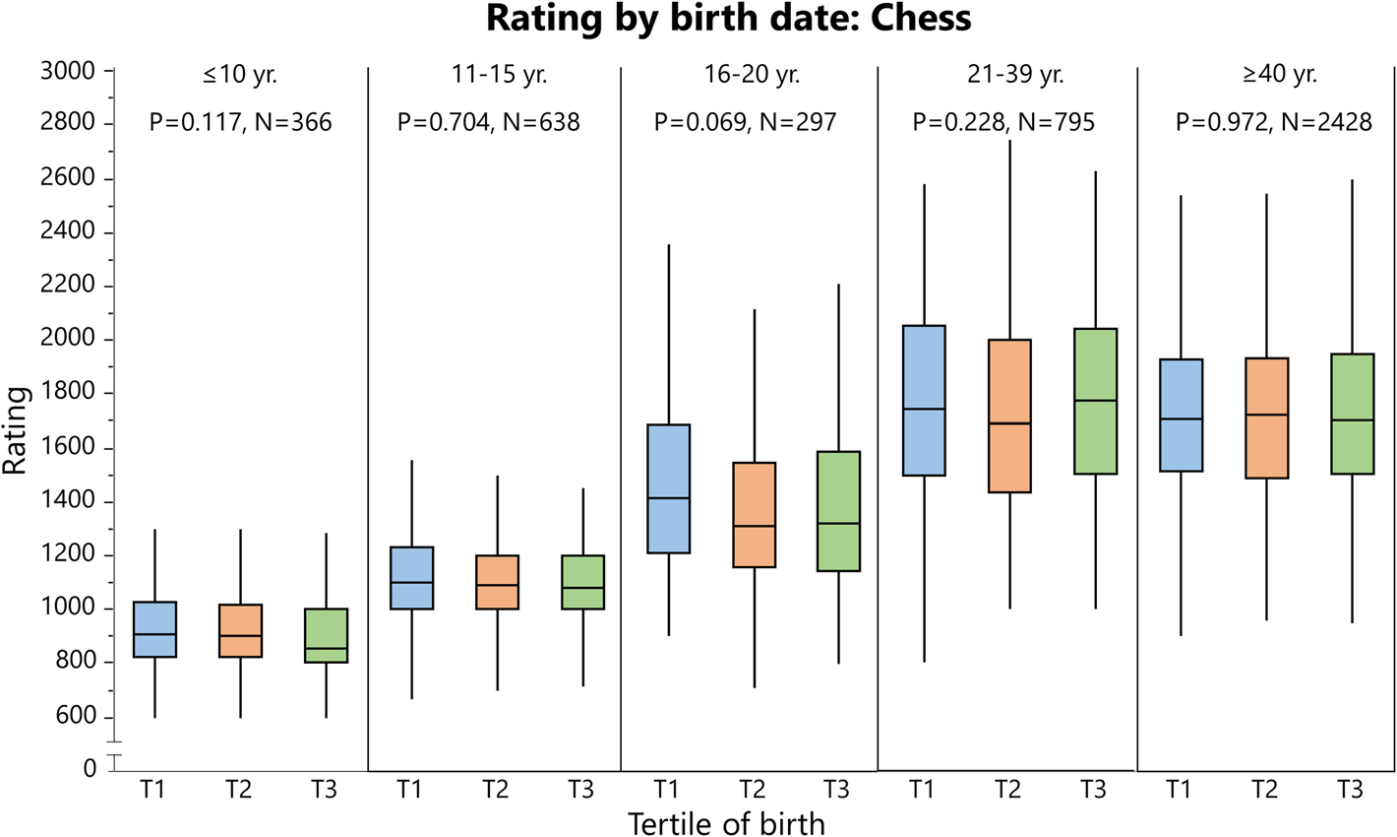
Rating (i.e., higher is better) for Swedish chess players analyzed by birth tertile (T) in different age groups with Kruskal-Wallis one-way analysis of variance (statistics at the top of the figure). For this figure, both sexes are pooled (similar rating points between sexes). The boundary of the box closest to zero indicates the 25th percentile, the line within the box marks the median, and the boundary of the box farthest from zero indicates the 75th percentile. End of whiskers represents the lowest and highest data point within 1.5× interquartile range of the first and third quartile

**Fig. 11:**
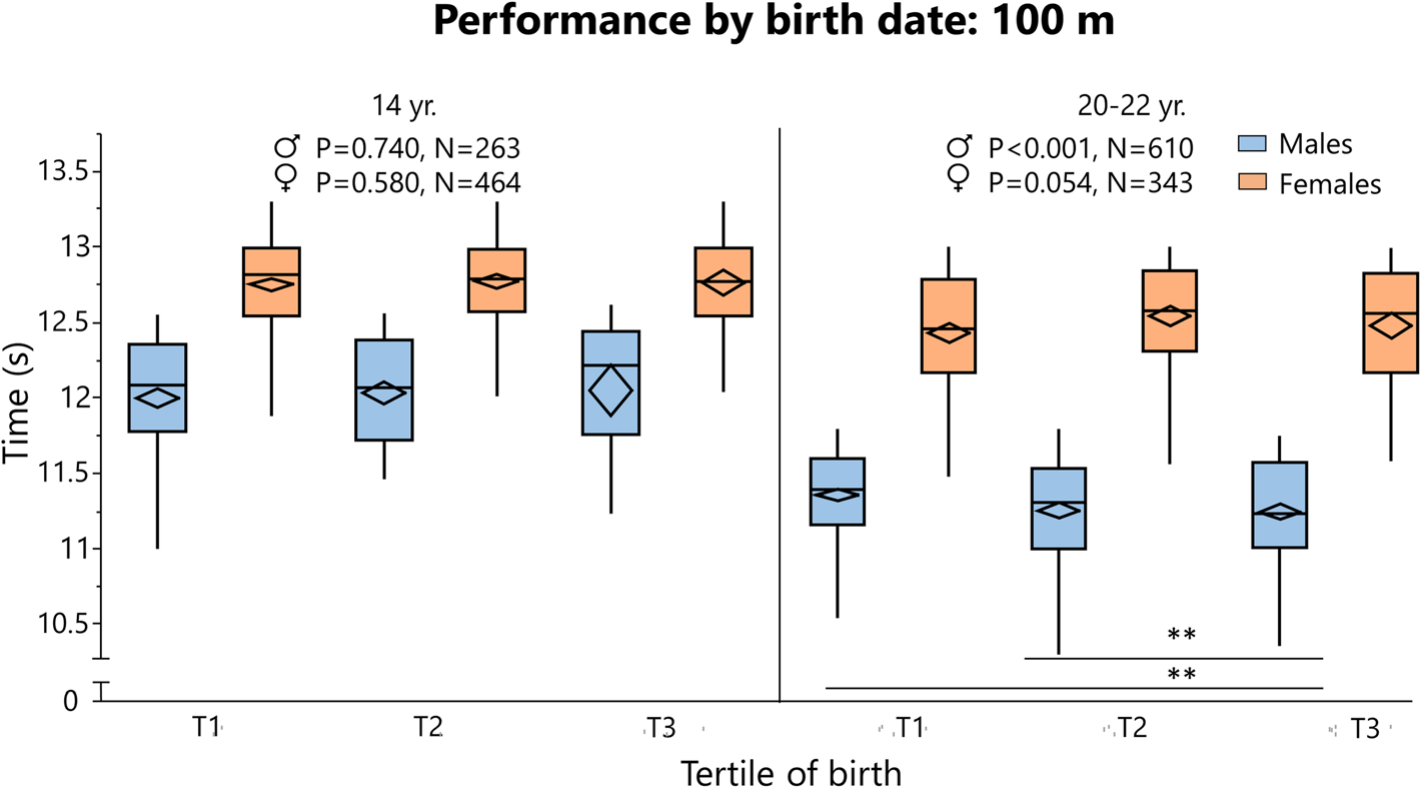
Performance for Swedish athletes in 100 m, analyzed by birth tertile (T) and sex in different age groups with one-way analysis of variance (statistics at the top of the figure). When significant followed by Tukey’s post-hoc test for multiple comparison between groups. The boundary of the box closest to zero indicates the 25th percentile, the line within the box marks the median, the confidence diamond contains the mean and the upper and lower 95% of the mean, and the boundary of the box farthest from zero indicates the 75th percentile. End of whiskers represents the lowest and highest data point within 1.5× interquartile range of the first and third quartile. **P ≤ 0.01

**Fig. 12:**
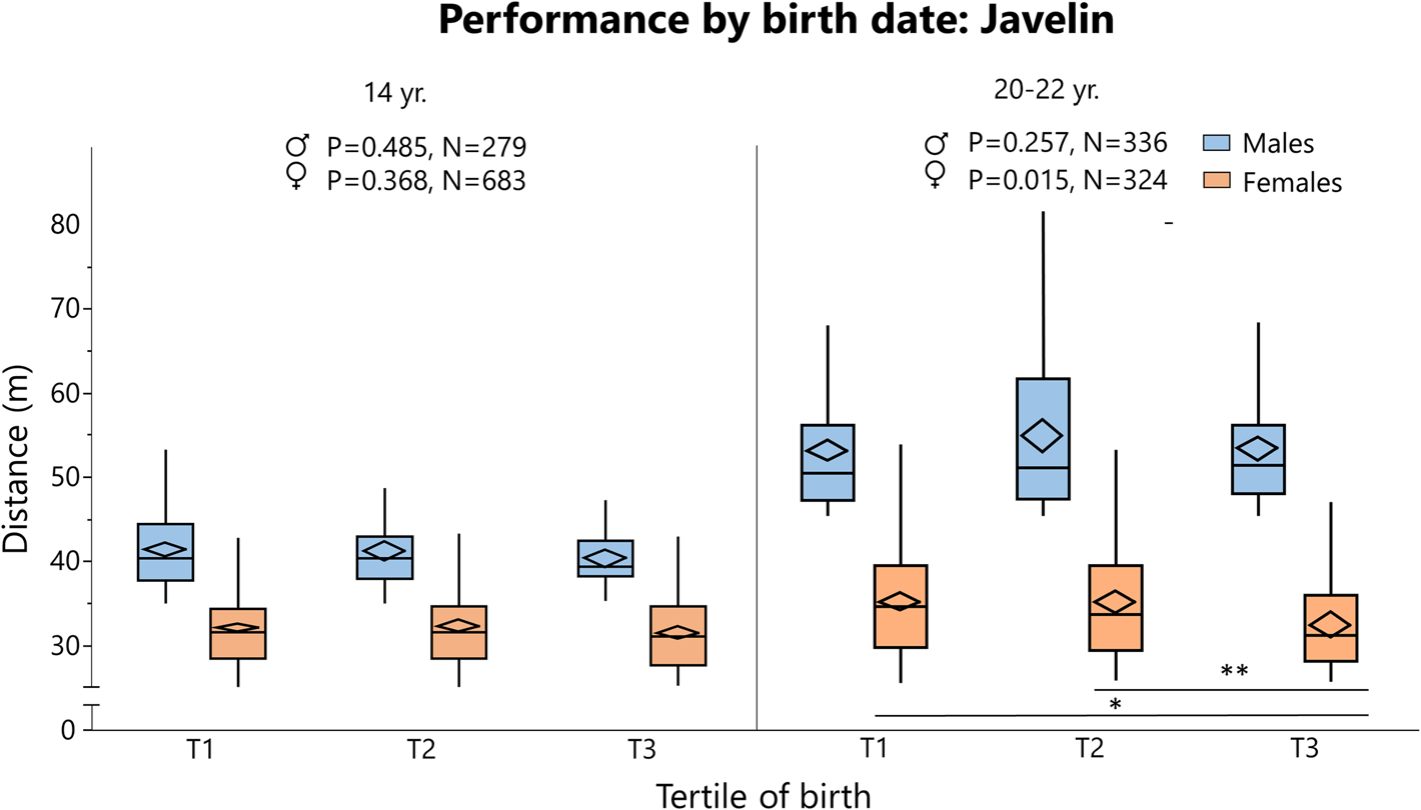
Performance for Swedish athletes in javelin, analyzed by birth tertile (T) and sex in different age groups with one-way analysis of variance (statistics at the top of the figure). When significant followed by Tukey’s post-hoc test for multiple comparison between groups. The boundary of the box closest to zero indicates the 25th percentile, the line within the box marks the median, the confidence diamond contains the mean and the upper and lower 95% of the mean, and the boundary of the box farthest from zero indicates the 75th percentile. End of whiskers represents the lowest and highest data point within 1.5× interquartile range of the first and third quartile. *P ≤ 0.05. **P ≤ 0.01

Rating (cross-country skiing and chess) and ranking (orienteering) data are summarized in Tables 6 and 7, respectively, and depicted in Figs. 8, 9 and 10. Performance data for 100 m and javelin are summarized in Tables 8 and 9, respectively, and depicted in Figs. 11 and 12. Data on 1500 m is found in Supplement 4.

**Table 6:** Rating by age group, sex, and birth tertile in cross-country skiing and chess

Sport	Age	Sex	Kruskal-Wallis		Steel-Dwass		Rating (median ± IQR)		
			*Χ*^**2**^	***P*** value	Levels	***P*** value	T1	T2	T3
Cross-country skiing^2^	≤ 10	Boys	6.1	0.048	T1–T3	0.052	400 ± 79.3	375 ± 84.0	378 ± 78.0
		Girls	1.9	0.391	–	–	376 ± 52.5	361 ± 74.0	362 ± 87.3
	11–15	Boys	15.7	< 0.001	T1–T2	0.018	499 ± 122.0	477 ± 109.0	469 ± 117.3
					T1–T3	< 0.001			
		Girls	3.4	0.185	–	–	448 ± 128.5	445 ± 106.5	429 ± 125.8
	16–20	Men	0.0	0.965	–	–	722 ± 162.5	737 ± 166.0	722 ± 210.0
		Women	0.1	0.995	–	–	615 ± 197.0	601 ± 180.0	607 ± 185.0
	21–39	Men	0.9	0.644	–	–	677 ± 230.0	686 ± 212.3	673 ± 219.5
		Women	0.9	0.652	–	–	592 ± 229.0	629 ± 260.5	586 ± 253.8
	≥ 40	Men	1.7	0.425	–	–	548 ± 152.5	527 ± 145.0	534 ± 136.5
		Women	0.5	0.796	–	–	474 ± 83.8	446 ± 171.0	475 ± 125.0
Chess	≤ 10	Boys	5.1	0.078	–	–	910 ± 220.0	896 ± 192.0	860 ± 200.0
		Girls	0.2	0.929	–	–	896 ± 312.0	900 ± 219.3	850 ± 208.0
	11–15	Boys	0.3	0.872	–	–	1100 ± 217.0	1100 ± 223.8	1080 ± 223.0
		Girls	7.8	0.020	T1–T2	0.018	1102 ± 276.0	1020 ± 159.3	1072 ± 169.0
	16–20	Men	2.9	0.233	–	–	1392 ± 495.3	1335 ± 416.3	1322 ± 405.0
		Women	9.1	0.011	T1–T2	0.019	1498 ± 383.8	1121 ± 252.8	1290 ± 311.3
	21–39	Men	4.0	0.138	–	–	1760 ± 542.8	1683 ± 590.0	1802 ± 540.0
		Women	2.0	0.375	–	–	1565 ± 751.8	1738 ± 391.5	1599 ± 267.5
	≥ 40	Men	0.0	0.991	–	–	1712 ± 408.0	1725 ± 443.0	1705 ± 442.0
		Women	0.5	0.797	–	–	1344 ± 972.0	1408 ± 750.5	1245 ± 548.0

*Χ*^2^ chi-squared statistic, *IQR* interquartile range, *T* tertile

Levels “T1–T3” means that T1 was better performing then T3

**Table 7:** Ranking age group, sex and birth tertile in Swedish orienteering

Age	Sex	Kruskal-Wallis		Ranking median ± IQR		
		***Χ***^**2**^	***P*** value	T1	T2	T3
11–15	Men	0.40	0.817	4117 ± 4206.8	4220 ± 3584.8	3865 ± 3031.5
	Women	0.33	0.847	2072 ± 2553.0	2160 ± 1865.0	2141 ± 1400.3
16–20	Men	0.97	0.615	2279 ± 4658.0	2918 ± 4279.0	3113 ± 4140.0
	Women	0.15	0.928	1718 ± 2303.0	1741 ± 2266.5	1691 ± 2445.8
21–39	Men	0.36	0.834	4827 ± 4935.8	4778 ± 5009.8	4689 ± 5179.0
	Women	0.45	0.800	2740 ± 2722.0	2777 ± 2585.0	2760 ± 2399.0
≥ 40	Men	0.93	0.636	4038 ± 3873.8	4010 ± 3693.0	4078 ± 3761.8
	Women	0.87	0.649	2322 ± 2062.0	2257 ± 2041.0	2233 ± 2241.0

*Χ*^2^ chi-squared statistic, *IQR* interquartile range, *T* tertile

**Table 8:** Performance in 100 m by age group, sex, and birth tertile Swedish athletics

Age	Sex	ANOVA			Tukey’s		Result(s) mean ± SD		
		***N***	F ratio	***P***	Levels	***P***	T1	T2	T3
14	Male	263	0.30	0.740			11.98 ± 0.47	12.03 ± 0.34	12.05 ± 0.46
	Female	560	0.55	0.580			12.74 ± 0.36	12.77 ± 0.32	12.76 ± 0.36
15	Male	464	0.38	0.684			11.55 ± 0.35	11.57 ± 0.37	11.53 ± 0.43
	Female	726	0.49	0.614			12.78 ± 0.32	12.8 ± 0.3	12.77 ± 0.33
16	Male	914	5.41	0.005	T3−T1	0.019	11.49 ± 0.28	11.55 ± 0.26	11.48 ± 0.29
					T3−T2	0.011			
	Female	816	1.78	0.169			12.72 ± 0.29	12.75 ± 0.28	12.76 ± 0.31
17	Male	993	3.18	0.042	T2−T3	0.047	11.51 ± 0.26	11.47 ± 0.31	11.53 ± 0.27
	Female	670	1.14	0.319			12.70 ± 0.30	12.74 ± 0.28	12.74 ± 0.29
19	Male	1116	1.76	0.173			11.46 ± 0.28	11.44 ± 0.3	11.42 ± 0.31
	Female	565	1.06	0.346			12.64 ± 0.32	12.64 ± 0.29	12.59 ± 0.32
22	Male	610	7.43	< 0.001	T3−T1	0.003	11.36 ± 0.30	11.26 ± 0.36	11.25 ± 0.33
					T2−T1	0.005			
	Female	343	2.95	0.054			12.43 ± 0.38	12.54 ± 0.35	12.48 ± 0.38
≥ 23	Male	196	2.06	0.130			11.35 ± 0.32	11.36 ± 0.35	11.24 ± 0.38
	Female	88	1.66	0.196			12.50 ± 0.39	12.57 ± 0.30	12.40 ± 0.31

*ANOVA* analysis of variance, *SD* standard deviation, *T* tertile

Levels “T3−T1” means that T3 was better performing then T1

**Table 9:** Javelin performance by age group, sex and birth tertile in Swedish athletics

Age	Sex	ANOVA			Tukey’s		Result (cm) mean ± SD		
		***N***	F ratio	***P***	Levels	***P***	T1	T2	T3
14	Male	279	0.73	0.485			4134 ± 490	4120 ± 561	4032 ± 340
	Female	683	1.00	0.368			3204 ± 489	3228 ± 554	3146 ± 412
15	Male	441	0.08	0.927			4439 ± 598	4431 ± 510	4461 ± 570
	Female	823	0.53	0.591			3126 ± 459	3124 ± 483	3094 ± 432
16	Male	462	1.43	0.241			4656 ± 734	4716 ± 653	4561 ± 580
	Female	1070	0.35	0.702			3126 ± 474	3124 ± 545	3094 ± 547
17	Male	500	0.23	0.794			4791 ± 729	4754 ± 661	4813 ± 734
	Female	712	5.07	0.007	T1–T3	0.0451	3203 ± 531	3250 ± 564	3084 ± 512
					T2–T3	0.0056			
19	Male	610	0.82	0.443			5000 ± 676	5079 ± 777	5069 ± 747
	Female	688	2.90	0.056			3276 ± 521	3321 ± 690	3177 ± 545
22	Male	336	1.36	0.257			5276 ± 777	5444 ± 920	5296 ± 679
	Female	324	4.23	0.015	T1–T3	0.0174	3478 ± 666	3476 ± 700	3206 ± 624
					T2–T3	0.0313			
≥ 23	Male	295	0.82	0.440			5219 ± 778	5151 ± 653	5082 ± 694
	Female	174	2.59	0.078			3333 ± 700	3182 ± 580	3065 ± 585

*ANOVA* analysis of variance, *SD* standard deviation, *T* tertile

Levels “T1–T3” means that T1 was better performing then T3

In cross-country skiing^2^, earlier born boys aged 11–15 years were higher rated than later-born peers, while there was no difference in other age groups. When pooling all age groups, T1 had a significantly higher rating than T3 in males (*P* = 0.026, Supplement 4). In orienteers, there was no difference in ranking between tertiles in any age group. In chess, the only differences were found in female young adolescents and female adolescent, where T1 was better ranked than T2. For athletics, there was no difference in performance for adults in 100 m, 1500 m (Supplement 3), or javelin. In 100 m, athletes born in T3 performed better than earlier born peers for 16- and 22-year-old males. In javelin performance, there was no difference between tertiles in males, while T3 athletes were inferior to T1 and T3 athletes in some female age groups (Table 9).

## Discussion

The present study is one of the most comprehensive on the relative age effect in sports, also investigating birthdates in relation to sports performance. The objective was to determine the magnitude and prevalence of RAEs in different individual sports, by age groups and sex, and in relation to physical performance. Our results show that RAEs are consistently prevalent in most individual sports in Sweden, both physically demanding and cognitive/skill-based. In general, no correlation between birthdate and performance was observed, neither in elite nor in recreational athletes. Findings of skewed birthdate distribution in all athletic populations should be viewed from both sports and health perspectives, and in light of the United Nations’ CRC.

### Subgroup Analyses

When stratified by age group, greater magnitude (distribution skewness, expected/observed ratio, and effect sizes) of RAEs was seen in the youngest (≤ 6 years; *V* = 0.31) and lower in older (≥ 40 years; *V* = 0.06) age categories. These findings are in line with previous research [35, 43], proposing a decline in the influence of maturation and growth with increased age, and must be a result either of participants born early in the season leaving sports at a later age, or a re-entry of participants with later birthdates.

In older adults (≥ 60 years), and even stronger in ≥ 65 years, an inversed RAE was found in male cross-country skiing^2^ and orienteers of both sexes. This can potentially be an effect of late-born individuals returning to sports later in life, after not having an equal chance against their early-born peers during childhood and adolescents. In the youngest age groups (< 10 years), multiple environmental constraints may influence children’s participation in sports [48], including parents’ reluctance to enroll later-born/maturing children [16, 49], more so with higher socioeconomic status [50]. It can be a fair assumption that among the youngest, other factors than the children’s will causes the pronounced RAE [16, 48–50]. At this young age, parents most likely decide when, where, and how the child participates in activities. In adolescence, selection is more often done by coaches.

When pooling sport types (Table 3), RAEs are significant in both physical sports and skill-based sports (*V* = 0.23 and 0.09, respectively) but with an important difference: The RAE in skill-based sports is inversed, with a slight overrepresentation in T3 (ratio = 1.02, supplementary material 4). This is mostly explained by male adults (21–39 years), which is by far the largest cohort in the skill-based sport types, and the only subgroup with an inversed RAE. Even distributions or inversed RAEs have been found in other non-physical skill-based sports, such as shooting [42, 51]. While not investigated in this study, sports with a high degree of technical skills and esthetics (such as gymnastics, dance, and figure skating) have been found to have no, inversed, or atypical RAEs [39, 52] discussed in detail by others [39, 48, 52, 53]. One possibility is that relatively younger individual’s dropout from physical sports, to focus more on studies or other hobbies, is a parental decision when the child lags behind in performance. Potentially, this could influence the inverse RAE in some E-sports cohorts. Meanwhile, E-sports today is possibly more competitive than 10–20 years ago, and in youths, being relatively older could be a competitive advantage. Speculatively, this could influence the RAE in youths compared to the inversed RAE in adults.

In the present study, RAEs were most prominent in physical sports such as cross-country skiing (both recreational and elite) and athletics, where T1 constituted of up to 51% of the athletes in some sub-samples, with large effect sizes, for instance, cross-country skiing boys ≤ 8 years (T1 = 51%, *V* = 0.43) for 14-year-old athletes in athletics, boys (T1 = 51%, *V* = 0.45) and girls (T1 = 48%, *V* = 0.41). In orienteering, RAEs were significant, but less skewed compared to cross-country skiing and athletics. In the relatively small sample of elite alpine skiers, a RAE was seen only in adult males, in line with previous research demonstrating RAEs in males only [54] or in both sexes [39] (Supplement 2).

It is generally assumed that the magnitude of the RAE in female sports is smaller due to less intense competition among young girls [42, 55]. Some have suggested that female sports are less strength-related than male sports, making the maturation-related developmental lead less decisive [56]. In the present study, RAEs were consistently seen in both sexes, and we did not observe a smaller overall RAE magnitude in females compared to males, as reported in some previous studies [20, 35, 51]. It has been suggested that relatively older female athletes may be at a greater risk of dropping out of sports [48], possibly due to early maturation that has been associated with increased negative psychosocial outcomes [57]. Further, some propose that a stereotyped definition of femininity could discourage early maturing females from participating in sports, in order to conform to socially constructed gender roles [58]. However, this is not reflected in our results were RAEs are consistently seen in both sexes.

Because most samples included a mix of recreational, competitive, and elite athletes, no sub-analysis was done on the level of competition. However, alpine skiers and cross-country skiing dataset 3 constituted of elite athletes only. As mentioned, alpine skiers showed no RAE when both sexes are pooled, in either junior or senior athletes. Only in adult males, a significant RAE was seen, with a large effect size but a limited sample size (*N* = 70, *V* = 0.39). In elite cross-country skiers, however, RAEs were prevalent in both junior and senior athletes (Fig. 4) as also seen in previous studies [39].

### Performance

Our results show that earlier born children sometimes perform better and are higher ranked than later-born peers. This trend is not seen in adult athletes where, in general, the average performance is the same, regardless of when you are born. There are just not as many athletes born late in the year. There is no surprise that children born earlier in the season also perform better—they are up to a year older than those born at the end of the season. In athletics, there is in general no difference between tertiles in performance (Figs. 11 and 12, Supplement 4). In some events and age groups, T3 and/or T2 actually performed better than T1. This is contrary to the general concept of RAE [59]. However, the result can be explained by the sample population. It is inevitable that the average level will be roughly the same for all three subgroups; all have been selected according to the same criteria (to place in the top 30). While the performance is equal, the distribution is nevertheless skewed. Hence, it is reasonable to say that the late-born adolescents that make the top 30 cut are early physically developed for their age in addition to being talented, while the early-born adolescents are overrepresented due to RAEs.

In this context, it has been shown that early sport success is not an adequate predictor of top-level performance [60]. Rather, entering competitions later is linked to better performance during adulthood [61]. The “underdog hypothesis” should be addressed in this context. The underdog hypothesis contends that, because they are less (physically) mature, relatively younger players must possess or develop superior technical, tactical, and physiological skills to be competitive [62]. In early adulthood, when differences in physical maturity are attenuated, this could in turn be in favor for the relatively younger athletes [63]. Importantly, for this hypothesis to be realized, later maturing/relatively younger athletes must be retained within the sport system. Todays’ early talent selection system, present in too many situations, is counter-productive when the goal is to find the very best future athletes.

### General RAE Discussion

Early talent selection, self-determined dropouts [64], or parents’ decision not to enroll relatively younger children in sports [48] all contribute to observed RAEs. With an increasing proportion of physical activity taking place in organized forms, such as in sports clubs [31] and the known relationship between childhood and adult physical activity levels [65], RAEs will impact long-term health. Also, being omitted from sports participation due to any of the above reasons violates the CRC, stipulating that the best interest of every child should always be in focus. Coaches’ and parents'selection of future elite athletes, annual age-grouping, systems, and adults’ administration of competition results must not be an excuse for the facilitation of sports dropout. Early selection and specialization in sports as young creates a requirement for children’s performance but is to a large extent a reflection of maturation, not talent and future performance. For those who do not live up to the requirements, there is a risk, or even guarantee, of being excluded from their team.

Should the RAE here seen in individual and previously in most team sports be disregarded because of the survival of the fittest? Nolan and Howell [66] state that the RAE will continue to exist and raise the question: “why shouldn’t it?” Why should it not be a Darwinian selection in sport at the highest level, and what says that the system of elimination must be fair? However, our results clearly demonstrate the lack of predictive power in early selection as athletes born early in the year do not perform better compared to their later-born peers. Most importantly, the current system results in discontinuation of physical activity in late-born children. In addition to the health benefits of sports participation, children who drop out of sports experience more social and emotional problems [67].

**Fig. 13:**
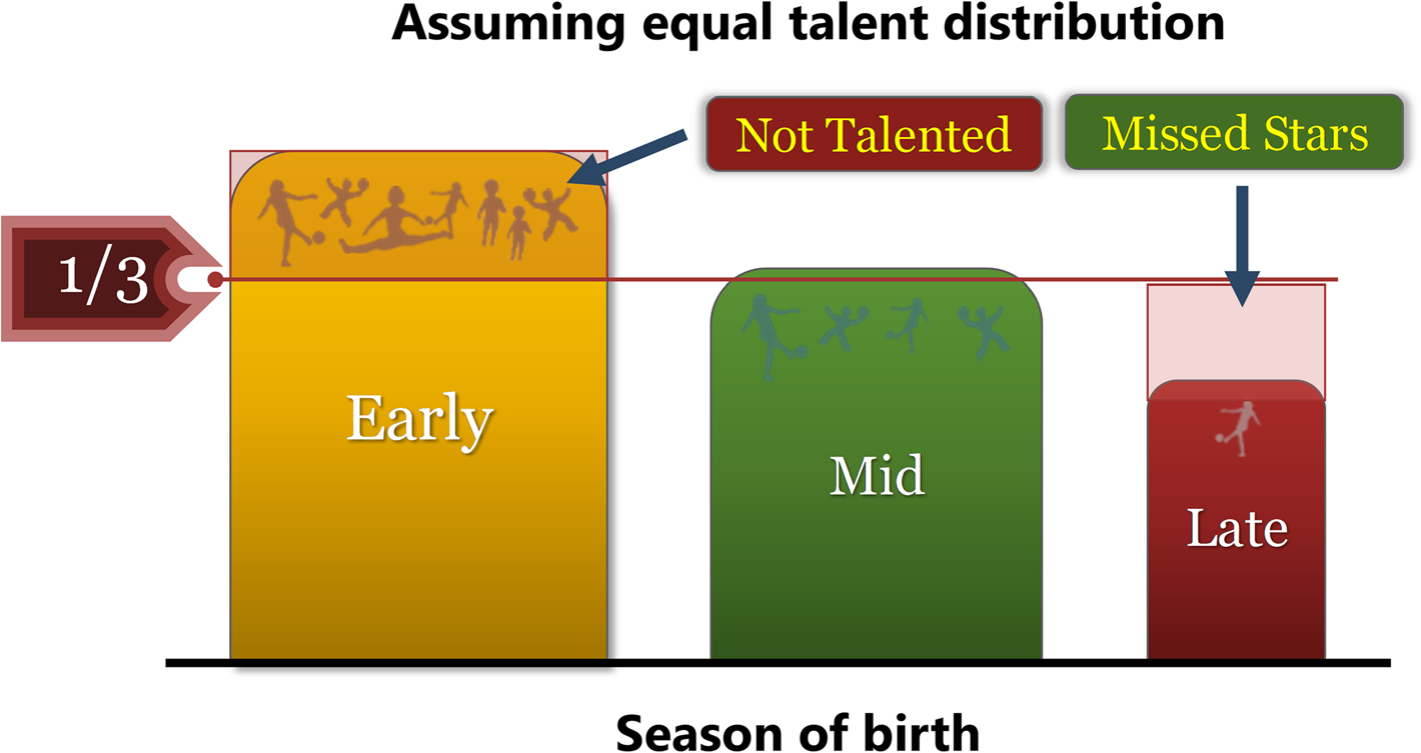
General distribution of athletes in individual sports in Sweden. Assuming equal talent distribution throughout the year, youth athletes are more likely to continue sports if they are born early. Consequently, some adult elite athletes reach elite level only because they were born early, when not truly talented. At the same time, late-born children, with the potential to become elite athlete, are missed stars due to early dismissal

It has also been appreciated that not everyone has the physical and mental capacities to make it to the top [68]. A fair organization in competitive sports is important for children to develop individually [7], but also for the very best senior athletes to-become not to be negatively selected at an early age in favor for individuals who do not have the future capacity to succeed as elite athletes. A skewed distribution based heavily on birthdate will eliminate future senior elite athletes while passing along the near elite. This can be viewed as the number of individuals above and below the expected distribution (Fig. 13). Assuming that talented children are born all year around, and not more likely in the spring, some individuals constituting the bars reaching above the expected distribution have made it to an elite level based on skewed selection due to birthdate. Other individuals who should have become elite athletes and contributed to the bars not reaching the expected distribution ever continued their athletic careers due to negative selection in younger years. At no time are all the physiologically and psychologically most suited athletes given the chance to reach their potential. Thus, it can be concluded that “survival of the fittest” in sports is skewed by less-than-optimal selection system and “fittest of the selected” a more appropriate term.

Moesch et al. [69] found that elite athletes pass important steps in their career at a significantly older age than their near-elite peers. Only after the age of 18 years will elite athletes complete more training hours in their sport than near-elite athletes, with a significant difference shown only after the age of 21 years. Also, elite athletes spend significantly fewer years on junior national teams, but more years on senior national teams. This, in combination with the conclusion made by Williams and Reilly [70], where it was seen as extremely difficult to identify those who will eventually reach the top, provides evidence that applying Darwinism on youths will inevitably lead to lower elite performance, as a group, among adult athletes.

To make competitions fair, to ensure that children are treated equally and ultimately develop optimal elite athletes, coaches and parents must be aware of the risk for skewed and inappropriate selections at young age. Loss of talent may be avoided if parents, coaches, and sport federations would allow prediction of success to occur at a later age [7, 27, 71]. Also, Martindale [68] concluded that de-emphasizing age-group success is a crucial concept to implement in talent development environment and that problems in selection and coaching will continue to exist if not a change in the stress on age group success is eliminated. The current system used in most countries may also disfavor early maturing children in the sense that they live under the impression that success can be reached without effort.

### Strengths and Limitations

A key strength of this study is that the distribution analyses are executed with the true parental distribution as a reference [46]. Also, the large sample size, often lacking in previous examinations, together with the multiple sports included results in a broad and robust examination of the topic. In line with similar research, multiple chi-squared tests have been executed, with no correction for multiple tests. A more stringent approach would reduce type I errors, i.e., false positives. Meanwhile, adjustment for multiple comparisons is not always desirable [72, 73]. The general result and conclusion would not be different, with a few less rejections of the null hypothesis (equal distribution of athletes).

## Conclusions

The RAE is present in most individual sports in Sweden and seen as early as in children age 3 to 6 years, as well as in older children, adolescents, and adults. A strive for early success eliminates potentially talented young athletes in team sports as well as individual sports. Youth sports should provide opportunities for individual performance development based on growth and maturity during adolescence. Age group squads should not be closed, but open, to provide later maturing athletes with the opportunity to become successful. Young athletes should be selected on skill and ability rather than on physical size. Further, it is necessary to educate leaders and coaches involved in youth sport about the importance, and the possible negative outcomes, of maturation-biased selection and to de-emphasize the importance of age-group success. Failing to address the relative age issue and the causing mechanisms may result in children discontinuing physical activity in general, creating a long-term negative effect on population health.

## Supplementary Information

**Additional file 1.** The Supplement file 1 can be accessed via the DOI: https://doi.org/10.6084/m9.figshare.12191898.

**Additional file 2.** The Supplement file 2 can be accessed via the DOI: https://doi.org/10.6084/m9.figshare.12191898.

**Additional file 3.** The Supplement file 3 can be accessed via the DOI: https://doi.org/10.6084/m9.figshare.12191898.

**Additional file 4.** The Supplement file 4 can be accessed via the DOI: https://doi.org/10.6084/m9.figshare.12191898.

**Additional file 5.** The Supplement file 5 can be accessed via the DOI: https://doi.org/10.6084/m9.figshare.12191898.

## Data Availability

All data generated or analyzed during this study are included in this published article and in Figshare data repository. DOI: 10.6084/m9.figshare.12191898

## Abbreviations

CRC: Convention on the Rights of the Child
RAE: Relative age effect
T: Tertile
*V*: Cramer’s *V*
*Χ*^2^: Chi-squared statistic
